# Cytodiagnosis of chondromyxoid fibroma

**DOI:** 10.4103/0970-9371.71873

**Published:** 2010-07

**Authors:** Vaishali A Walke, Suprita P Nayak, Maitreyee M Munshi, Sudhakar K Bobhate

**Affiliations:** Department of Pathology, Government Medical College, Nagpur, Maharashtra, India

**Keywords:** FNA, bone tumors, chondromyxoid fibroma

## Abstract

Chondromyxoid fibroma (CMF) is an unusual tumor that predominantly affects long bones of young adults. We present two cases of CMF that were diagnosed on cytology. The specific cytological features included varying combinations of chondroid, myxoid and fibroid elements. These features when correlated with clinico-radiological findings helped to arrive at a correct diagnosis. Thus a definitive diagnosis of CMF can be made on cytology based on which further line of treatment can be planned.

## Introduction

Fine-needle aspiration cytology (FNAC) is an established cost-effective diagnostic tool for skeletal lesions. The cytomorphological findings in conjunction with clinical and radiological features help not only in the initial diagnosis but also in its further categorization.[1,2] Many studies have elaborated on FNAC of bone tumors, but there are very few detailed reports regarding benign chondroid neoplasms.[3–6]

Chondromyxoid fibroma (CMF) is one such tumor that is characterized by incomplete cartilage differentiation. This tumor was first described as a distinct entity by Jaffe and Lichtenstein in 1948.[6,7] It represents <0.5% of all bone tumors and is the least common benign tumor of cartilaginous origin.[3,6–8] CMF is usually an intra-medullary eccentric lesion located in metaphyseal region of the distal femur and proximal tibia.[7,8] But its occurrence at other unusual sites is well documented, where it can cause diagnostic difficulties.[2,6,8,9] We present two cases of CMF that were diagnosed on cytology.

## Case Reports

### Case 1

**Figure 1:**
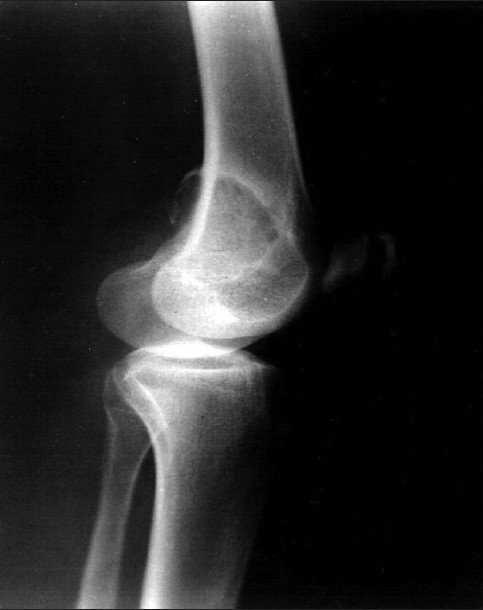
Radiograph showing, well-defined lytic, eccentric, expansile lesion with sclerotic rim in the epimetaphyseal region at the lower end of femur

A 22-year-old lady presented with a history of pain and swelling of left knee since 1 year. She also gave history of difficulty in moving the same joint. Radiograph showed a well-defined lytic, eccentric, expansile lesion with sclerotic rim in the epimetaphyseal region at the lower end of femur [Figure 1]. Clinical diagnosis was giant cell tumor of bone.

### Case 2

An 8-year-old girl presented with complaints of pain and swelling of left lower leg of 4 months duration. She also gave history of trauma at the same site. Radiograph showed an eccentric, expansile, lytic lesion at the metaphyseal region at upper end of tibia. Clinically the differential diagnoses included aneurysmal bone cyst and giant cell tumor of bone. FNAC was followed by curettage. FNAC was done in both the cases and smears were prepared. Wet fixed smears were stained with hematoxylin and eosin (H and E) and Papanicolaou stains, while air-dried smears were stained with May–Grünwald–Giemsa stain (MGG).

### Cytological findings

**Figure 2:**
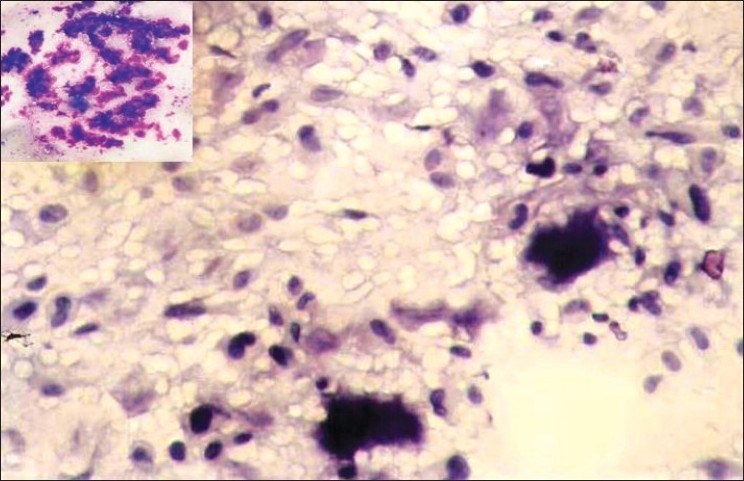
Smear showing polymorphous population of cells dispersed singly along with the matrix. Individual cells are round, oval to spindle with few cells showing binucleation and nuclear indentation (Pap, ×200). Inset: Abundant purple coloured, chondromyxoid matrix (MGG, ×100)

In both the cases, the smears revealed abundant material that was predominantly composed of chondromyxoid matrix with moderate cellularity. The cells were of variable sizes and shapes and were mostly dispersed singly in myxoid background [Figure 2]. Some of the cells were round to oval with well-defined borders, moderate cytoplasm and centric to eccentric round to ovoid nucleus. The chromatin was uniformly bland and no nucleolus was seen. Few chondroblast-like cells with indented nuclei were noted entrapped in the matrix. Occasional binucleate cells were observed. Spindle to stellate cells, scattered singly and in clusters were also noted. Few fibroblast-like spindle cells were seen embedded in the myxoid matrix. Mild nuclear pleomorphism was present but no mitotic figures could be identified. Few benign osteoclastic giant cells were seen in case 2.

The chondromyxoid matrix appeared as dense fibrillary irregular fragments, which was best appreciated in MGG-stained smears due to its magenta-pink to purple color [Figure 2]. No well-formed mature cartilaginous fragments with cells sitting in lacunae, or foci of calcification were noted in either of the cases. Correlating the above cytomorphology with the clinical and radiological details, cytological diagnosis of CMF was offered in both the cases. Bone curettage samples of both the cases were received and sections were prepared for histopathological examination.

**Figure 3:**
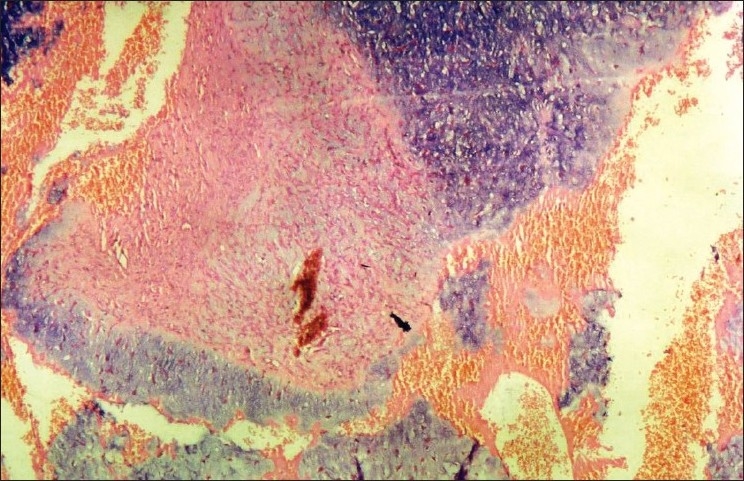
Section showing pseudo-lobules of myxoid and chondroid tissue separated by zones of fibrous tissue (H and E, ×200)

In both the cases, sections studied showed pseudo-lobules of myxoid and chondroid tissue separated by zones of fibrous tissue [Figure 3]. The cells were oval, spindle to stellate with small oval nuclei and uniform dense chromatin. No mitotic figures were seen. Areas of increased cell density forming fibrous cap were noted at the periphery of lobules. Well-formed hyaline cartilage, calcification or new bone formation was not evident. Thus CMF was confirmed on histopathology.

## Discussion

CMF is a rare benign chondroid tumor that still causes diagnostic difficulties when seen at unusual locations.[6–8] Prior to its segregation as a distinct tumor, its rarity coupled with focal hypercellularity and cellular polymorphism caused it to be mistaken for chondrosarcoma.[6,9] However, the benign course of this neoplasm makes an accurate pre-operative diagnosis crucial so as to avoid debilitating and unnecessary surgical procedures. Previous reports have suggested that the cytological features of CMF are distinctive enough to make a confident pre-operative diagnosis on FNAC after clinico-radiological correlation.[3–5]

Clinically, CMF generally has a typical presentation. It usually affects the young with a peak incidence in the second and third decades of life and a slight male preponderance. The patients usually present with pain and swelling of long-standing duration. The sites of predilection are long tubular bones in about half the cases, particularly distal femur and proximal tibia, while in one third of cases flat bones, such as the ileum, are involved. Less common sites include ribs, vertebrae and the bones of skull and hands.[8,9]

Benign cartilaginous tumors almost always appear benign on radiology with sharp circumscribed borders, intact overlying periosteum and no adjacent soft tissue swelling. CMF is typically seen as a metaphyseal, oval, eccentric lesion ranging from 1 to 10 cm. The long axis of the tumor parallels the long axis of the bone with sharp sclerotic rim and rare calcification.[3,6]

Both the cases being presented had the typical clinical features of pain and swelling. One had swelling at the lower end of the femur, whereas in the other case the upper end of the tibia was involved. The radiographical findings in both the cases were also suggestive of benign lesions. Cytological findings of CMF have appeared in only a few reports so far.[2–5,8] The specific features being varying combinations of chondroid, myxoid and fibroid elements. The chondroid and myxoid elements form the irregular fragments of matrix in the background. The cellular elements comprise a variable mixture of spindle cells, stellate cells and chondroblasts with nuclear pleomorphism but with uniformly bland, smudgy chromatin and no mitotic figures.

The lesions that need to be considered in differential diagnosis of chondromyxoid fibroma on cytology range from benign tumors, such as chondroblastoma to sinister lesions, such as myxoid chondrosarcoma.[2,3,5]

Chondroblastoma characteristically is an oval, well-defined, epiphyseal lesion with sclerotic rim and matrix calcification, whereas CMF is a metaphyseal lesion with rare intra-tumoral calcification. On cytology, chondroblastoma is a cell-rich tumor in contrast to matrix-rich CMF. Three dominant components have been described in cytology smears of chondroblastoma. They include the neoplastic mononuclear cells (chondroblasts), multinucleate giant cells and chondroid matrix. Recognition of chondroblasts based on its cytological features has been emphasized as the diagnostic hallmark of this lesion along with chondroid matrix surrounding the individual cells. As against chondroblastoma, osteoclastic giant cells and calcification, though occasionally seen, are not prominent features of CMF.[3,6] CMF can also be mistaken for chondrosarcoma on cytology due to polymorphic cell composition and nuclear exuberance, especially the myxoid variant, due to the presence of chondroid and myxoid matrix in the background. However, chondrosarcoma characteristically shows fragments of hyaline cartilage and cells sitting in the lacuna. The individual cells can be uninucleate or binucleate showing nuclear pleomorphism and frequent mitosis. The radiological findings of cortical erosion, soft tissue extension and intra-tumoral calcification are also supporters for the diagnosis of chondrosarcoma as against CMF.[5,6]

Rare examples of malignant CMF and sarcomatous transformation of CMF have been reported. However, it is unclear whether malignant CMF actually exists or whether it represents another type of sarcoma that has been misdiagnosed.[6] The ideal treatment for CMF is curettage combined with bone grafting, and hence a correct pre-operative diagnosis is very helpful in selecting this line of management.

FNAC can thus prove to be an invaluable tool in the diagnosis of CMF. Although CMF can cause diagnostic difficulty because of its rarity and unusual morphology, yet the cytological features, in conjunction with the clinico-radiological findings, are distinctive enough for making a confident diagnosis on cytology.
